# Self-illumination of Carbon Dots by Bioluminescence Resonance Energy Transfer

**DOI:** 10.1038/s41598-019-50242-9

**Published:** 2019-09-24

**Authors:** Jisu Song, Jin Zhang

**Affiliations:** 1 School of Biomedical Engineering, University of Western Ontario, London, Ontario N6A 5B9 Canada; 2 Department of Chemical and Biochemical Engineering, University of Western Ontario, London, Ontario N6A 5B9 Canada

**Keywords:** Biological fluorescence, Nanobiotechnology

## Abstract

Carbon-dots (CDs), the emerging fluorescent nanoparticles, show special multicolor properties, chemical stability, and biocompatibility, and are considered as the new and advanced imaging probe in replacement of molecular fluorophores and semiconductor quantum dots. However, the requirement of external high power light source limits the application of fluorescent nanomaterials in bio-imaging. The present study aims to take advantage of bioluminescence resonance energy transfer mechanism (BRET) in creating self-illuminating C-dots. *Renilla* luciferase (Rluc) is chosen as the BRET donor molecule. Conjugation of *Renilla* luciferase and C-dots is necessary to keep their distance close for energy transfer. The optimal condition for achieving BRET is investigated by studying the effects of different factors on the performance of BRET, including the type of conjugation, concentration of carbon dots, and conjugation time. The linear relationship of BRET efficiency as a function of the amount of C-dots in the range of 0.20–0.80 mg/mL is observed. The self-illuminating carbon dots could be applied in bioimaging avoiding the tissue damage from the external high power light source.

## Introduction

Fluorescent nanoparticles (FNP), e.g. quantum dots, have been extensively studied and considered as an alternative imaging probes because their special size-dependent fluorescence, superior photostability, and high intensity^1^.

Carbon dots (CDs), the emerging FNP, show excitation-dependant emission, good biocompatibility, and chemical stability. Their special multicolor photoluminescence have attracted extensive attention because of the various applications in photovoltaic devices, bioimaging, etc^2,3^. The special fluorescence of CDs is complicated in nature and is subject to debate^4^. There has been many theories to explain the fluorescence of CDs: quantum size effect, which originates from the nanoscale size of CDs; degree of surface oxidation; and surface functional groups, such as carbonyls and imines^5^.

However, external high power light source is necessary to initiate the excitation of FNPs, which may result in photocytotoxicity and the damage of tissue^6^. There are several mechanisms in which fluorescence is achieved without external high power light source, for instance, chemiluminescence resonance energy transfer (CRET) and bioluminescence resonance energy transfer (BRET). Both involve a non-radiative energy transfer between a light energy donor and a luminescence acceptor. When the distance between donor and acceptor molecules is close, <10 nm, and the emission of the donor molecular is able to cover the excitation or absorption of the acceptor molecular, these luminescence resonance energy transfers can occur^7–9^. CRET depends on chemiluminescence reactions in order to excite the CRET acceptor molecule. In essence, chemiluminescence reaction results in an electronically excited product followed by the electron dropping to ground state while emitting photon to release energy^10^. While this successfully eliminates the need for external light source, new limitations are introduced. In order to lower the activation energy of the initial chemiluminescence reaction, metallic ions or catalyst enzymes are often used as catalysts^11^. Furthermore, cofactors may also be necessary for the initial reaction to take place. Biocompatibility may be jeopardized with the use of metallic ions; moreover, the required catalyst and cofactors may not be compatible with the microenvironment. Unlike CRET, BRET utilizes natural bioluminescent molecules found in firefly, gellyfish, and other organisms, as the light energy donor^12^. For instance, enzymatic reaction of *Renilla* luciferase (Rluc) with its substrate, coelenterazine (CTZ), can generate luminescence at 480 nm^13^. Because catalysts and cofactors are not required for the reaction to take place, BRET is more biocompatible compared to CRET. Recently, bioconjugation of bioluminescent proteins onto nanostructures, e.g. gold nanoparticles, semiconductor QDs, have been applied in biosensors and bioimaging without the external power supply^14–17^. Compared to the BRET pair containing the acceptor made of organic dye and/or other nanostructures, CDs could be particularly of interest in using for BRET pair, as they do not need extra steps to modify functional groups for further bioconjugation, and CDs demonstrate significantly better biocompatibility compared to other fluorescence nanostructures, e.g. quantum dots.

**Figure 1:**
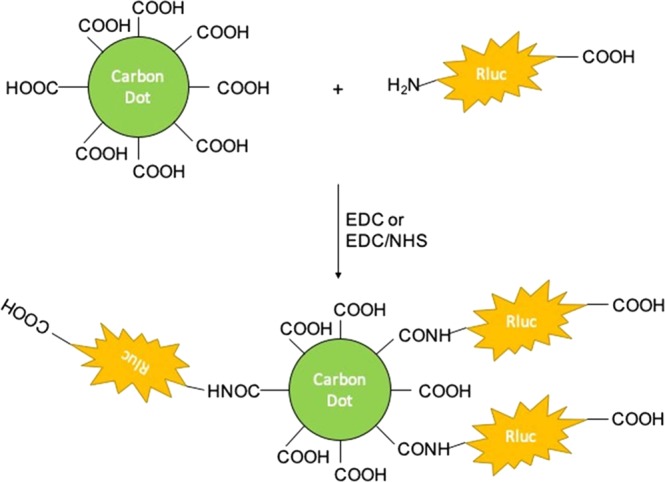
EDC- or EDC/NHS-mediated conjugation of bioluminescent protein, *Renilla* luciferase (Rluc) to CDs. Both conjugation couples the free surface carboxylic acids on carbon dots with the amine group on the N-terminal of Rluc. The close proximity between the two molecules allows for bioluminescence resonance energy transfer.

Very few studies have reported the self-illuminescence of CDs through bioconjugation with bioluminescence proteins. Herein, we demonstrate a method to produce the self-illuminated CDs by utilizing the BRET technique. Figure 1 illustrates the strategies on bioconjugation of Rluc onto CDs through carboxyl-to-amine crosslinking. Two major water-soluble carbodiimide crosslinkers, N’-(3-dimethylaminopropyl)-N-ethylcarbodiimide (EDC) and EDC associated with N-hydroxysuccinimide (NHS), were investigated. Other different factors including the ratio of CDs and Rluc and the bioconjugation reaction time, are investigated towards the successful BRET performance.

## Results

### Bioluminescence of renilla luciferase and photoluminescence of C-dots

**Figure 2:**
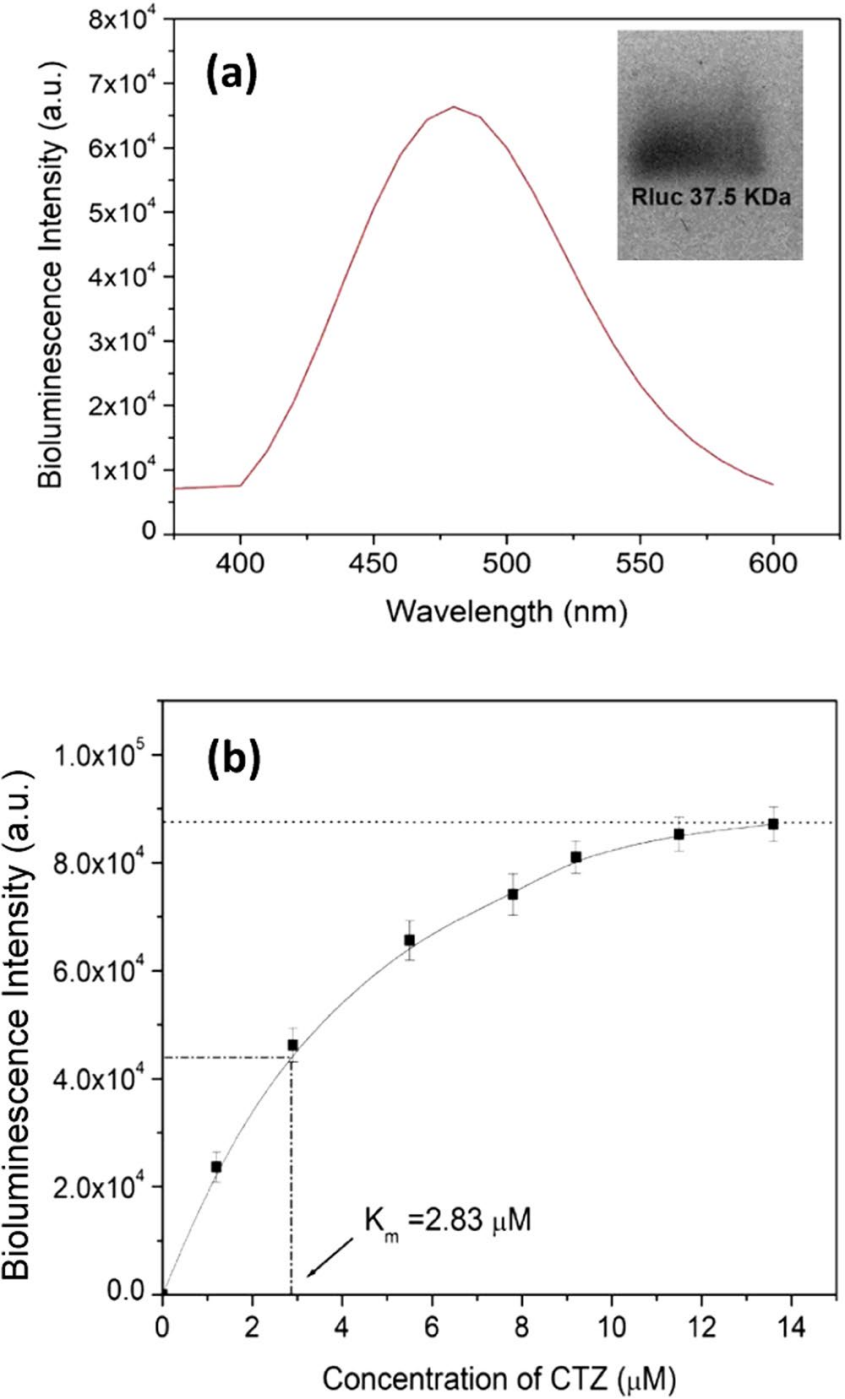
(**a**) Bioluminescence of 2 μM *Renilla* luciferase reacting with 7.8 μM CTZ. The small inset shows the purified Rluc on the SDS-PAGE gel. (**b**) Bioluminescence intensity as a function of the concentration of the substrate, CTZ.

The maximum bioluminescence intensity of Rluc (2 µM) is observed at 480 nm, which increases with increasing the amount of CTZ as shown in Fig. 2a. The purified Rluc is characterized by the agarose (1.2%) gel electrophoresis. Only 37.5 KDa Rluc shows on the SDS-PAGE gel (see the small inset in Fig. 2a). The bioluminescence intensity of Rluc (2.0 µM) increases linearly as the concentration of CTZ increase from 0 to 13.6 µM; while the bioluminescence intensity reaches a plateau when the concentration of CTZ increases up to 13.6 µM, which follows the Michaelis–Menten model. As the increase of bioluminescence intensity is corresponding to the reaction velocity, it is estimated that the Michaelis constant (K_m_) is around 2.83 µM, as shown in Fig. 2b. Therefore, 30 µM CTZ, the substrate, was used in reacting with 2 µM Rluc to generate bioluminescence, as it is normal to obtain the maximum reaction velocity to an enzyme-involved reaction by adding the substrate with10–20 times of K_m_.

**Figure 3:**
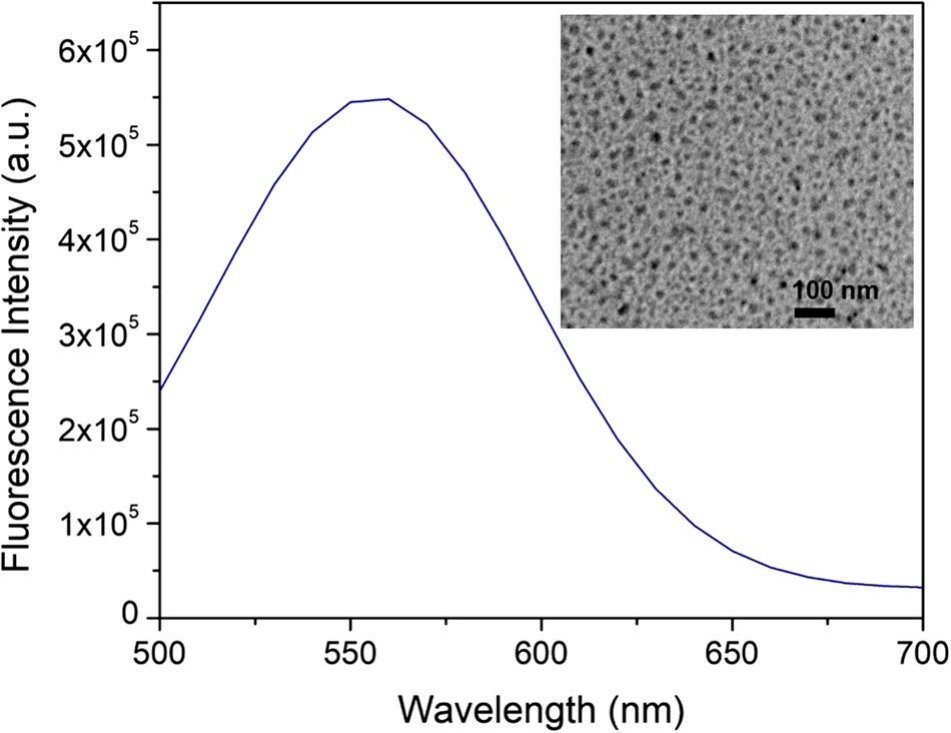
(**a**) TEM micrograph of CDs. (**b**) Photoluminescence of CDs under an excitation wavelength of 480 nm.

On the other hand, the CDs were measured by TEM as shown in the small inset of Fig. 3. Average particle size is estimated at 13 ± 3 nm. The photoluminescence of free CDs was measured by fluorospectrometer. The UV-vis absorption spectra of CDs with different concentration were shown in Fig. S1. All CDs samples in aqueous show broad absorption spectra with the same absorbance peaks centered at 270 nm, 340 nm, and 405 nm as the previous study on CDs^17^. The water-soluble CDs made by this microwave oven-assisted process has excitation-wavelength-dependent PL properties with maximum quantum yield about 14%^18^. As the emission of Rluc is centered at 480 nm, therefore, the PL of CDs was measured under the excitation, λ_ex_, of 480 nm. The maximum emission, λ_em_, of CDs is around 557 nm as shown in Fig. 3.

### Comparison of EDC- and EDC/NHS-mediated conjugations

Water soluble carbodiimide crooslinking agents, EDC and EDC/NHS, were employed to bioconjugate Rluc onto CDs by forming an amide bond. Different ratios of Rluc (2 μM) to CDs(0.10 μg/mL, 0.20 μg/mL, 0.40 μg/mL, 0.80 μg/mL, and 1.20 μg/mL) were studied, respectively; all other factors, such as carbon dot concentration and conjugation time, were kept constant. Compared to EDC-mediated conjugation, all samples for EDC/NHS-mediated conjugation lost fluorescent signals; there was a lack of any peaks to indicate bioluminescence.

### The factors on the performance of BRET

**Figure 4:**
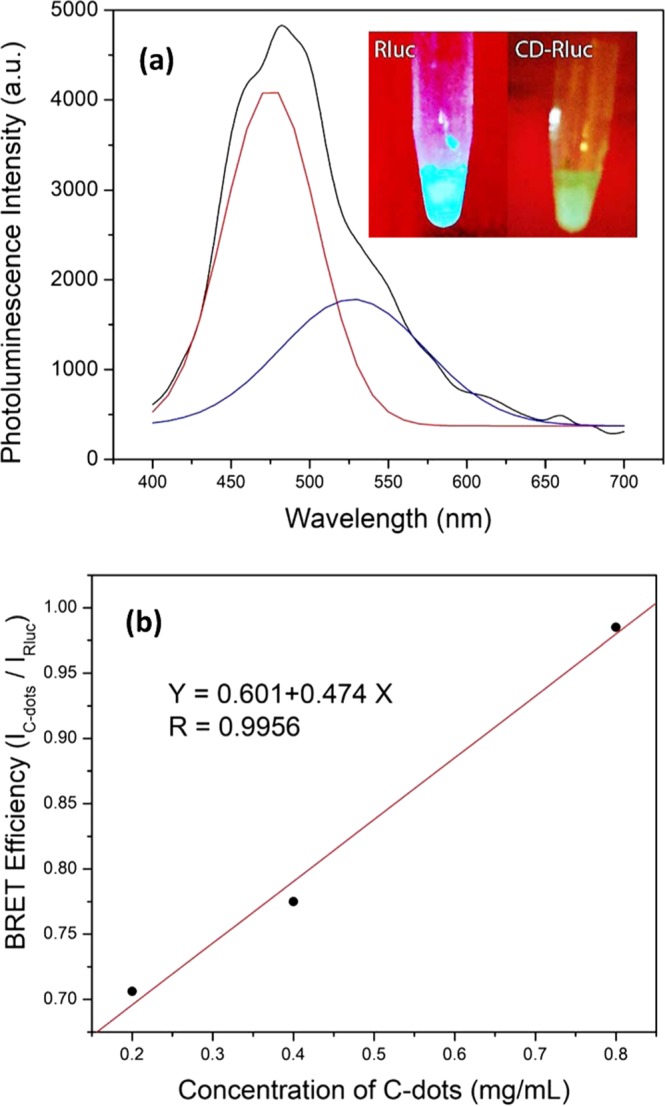
(**a**) BRET spectrum of sample made through EDC-mediated conjugation of 0.20 mg/mL CDs and 2 μM Rluc for 6.5 hours. The black line represents the BRET profile of the sample; the red line and the blue line obtained by using multi-peak fitting are attributed to the bioluminescence of Rluc and the photoluminescence of CDs, respectively. The small inset is the photo of the 2 μM Rluc and CD-Rluc compex (i.e. 0.20 mg/mL CDs conjugated with 2 μM Rluc). (**b**) BRET efficiency as a function of CDs concentration.

Three incubation time periods for the bioconjugation of Rluc to CDs were studied: 1 hr, 6.5 hr, or 24 hr. Generally, for all incubation times, the samples with the lowest concentration of CDs only exhibited one peak around 480 nm, representing the bioluminescence of Rluc without observable BRET phenomenon. Similarly, the samples with the highest concentration of carbon dots also demonstrated a single peak attributing to the bioluminescence of Rluc during earlier conjugation time frames, however lost photoluminescence for longer conjugation times. BRET phenomenon can be observed when the concentrations of CDs is in the range of 0.20 mg/mL to 0.80 mg/mL. It is also observed that BRET efficiency is dependent on the conjugation time. Within the first hour of bioconjugation, only the bioluminescence of Rluc can be observed with the lack of BRET phenomenon. When incubation time increases to 6.5 hours, BRET signals could be observed for samples containing 0.20 mg/mL, 0.40 mg/mL, and 0.80 mg/mL of CDs. Figure 4 is the BRET profile of the bioconjugation of 2 μM Rluc and 0.20 mg/mL CDs. The BRET profile (black line) of the sample is shown in Fig. 4a; the red line and the blue line obtained by using multi-peak fitting are attributed to the bioluminescence of Rluc and the photoluminescence of CDs, respectively. The small inset of Fig. 4a is the photos of 2 μM Rluc and CD-Rluc complex in which 2 μM Rluc is reacted with 0.20 mg/m CDsL Please note the photos taken without external high power light supply. Whereas our previous study on fluorescence nanoparticles indicates that a photoluminescence spectrometer used to generate an excitation in the range of near-infared is required a power larger than 0.5 W^19^. Rluc is bright blue. With the conjugation of CD (which emits within the yellow-orange colour), we can observe the emission colour becoming more green from the mixture of pure Rluc and CD-Rluc complex. The brightness of bioluminescence is also compromised however with the conjugation of CD, which is unsurprising as some population of Rluc would have lost its function from incorrect conjugation at the lysine residue. Fig. 4b is the BRET efficiency increasing with increasing the amount of conjugated CDs from 0.20 mg/mL to 0.80 mg/mL.

## Discussion

Clearly, bioconjugation is vital for achieving the self-illuminated CD by using BRET mechanism. Although NHS is considered to enhance the crosslinking efficiency of EDC in integrating bioluminescence molecules and nanostructures by forming amide bond, our study indicates that a complete loss of bioluminescence occurs when using EDC/NHS crosslinking. It is noted that both EDC- and EDC/NHS-crosslinked conjugation is non-selective in that the coupling agents conjugate any free carboxylic acid with any free amine group. Due to their non-selective nature, conjugation usually occurs between the C-terminal of one protein to the N-terminal of another but also between C-terminal of one protein and amine on a lysine residue of another protein^20^. A previous study had shown that conjugation at lysine residues may be fatal to protein structure for smaller proteins and can result in loss of function^20^. Rluc is a small protein with molecular weight of 36 kDa, which may have lead to a similar loss of function as seen in previous literature^21,22^. Furthermore, another literature had studied the structure of Rluc and discovered that mutations in certain lysine residues result in decreased function^23^. The conjugation of CD to the amine group of lysine may have led to loss of structurally important intermolecular forces resulting in the loss of function.

Although the loss of function was more pronounced in EDC/NHS-mediated conjugation, we believe that the loss of bioluminescence due to the high concentration of CDs (>0.80 mg/mL) and long conjugation time (~24hrs) originates from the same problem. With higher concentrations of CDs, more CDs would have been available to conjugate with amines, which increases the probability of conjugation at lysine residue. Similarly, with longer conjugation time, CDs would have more opportunity to conjugate with the amines, which would also increase the probability of conjugation at lysine residues. Even though EDC is less effective coupling agent by itself, with high enough concentration and long enough time, conjugation at lysine residues are not preventable.

On the other hand, no BRET phenomenon is observed for low concentration of C-dots and short conjugation time (~1 hour). This can be explained by the kinetic nature of bond formation. For bonds to form, molecules must collide with one another at a correct angle. With low concentrations of C-dots, the probability of collision decreases, and with short reaction times, the number of collisions is bound to also decrease.

BRET phenomenon is observable for 0.20–0.80 mg/mL of C-dots conjugated with 2 μM Rluc by using EDC for 6.5 hours. The BRET efficiency (E) is calculated by the following equation (Eq. 1)^17^

1 $${{E}}_{{BRET}}=\frac{{{I}}_{{CDs}}}{{{I}}_{{Rluc}}},$$

where *I*_*CDs*_ is the integrated emission within the range of 450–650 nm from CDs caused by the BRET mechanism, *I*_*Rluc*_ the integrated emission within the range of 390–550 nm from Rluc. Figure 4b indicates that the BRET efficiency increases linearly with increasing concentration of CDs from 0.20 mg/mL to 0.80 mg/mL as sh. Higher BRET efficiency signifies better energy transfer from Rluc to CDs^17,24^. BRET efficiency increases with increasing CD concentration since more conjugation would take place; however, as mentioned earlier, when the concentration of CDs increases beyond 1.20 mg/mL, the bioconjugation at lysine becomes fatal to protein function. Previous studies on BRET effect of organic dyes conjugating with bioluminescence protein, e.g. Rluc, show that nonspecific BRET signals tend to increase linearly with increasing acceptor concentrations^25–27^.

## Conclusions

In summary, water-soluble CDs with average particle size of 13 ± 3 nm were produced by a microwave oven-assisted process. To achieve the illumination of C-dots without external high power light supply, bioluminescence protein, Rluc, was conjugated onto the CDs. The bioluminescence of Rluc is at 480 nm, which is able to excite the photoluminescence of CDs through the BRET mechanism and generate emission of CDs at 557 nm. The effect of the concentration of the substrate, CTZ, on the bioluminescence was investigated. It was estimated that the Michaelis constant (K_m_) is around 2.83 µM to the enzyme reaction of 2 µM Rluc. Different factors, such as crosslinking agents, ratio of Rluc to C-dots, and the incubation time for the bioconjugation, have been investigated to achieve high BRET efficiency. Our study indicates that the BRET efficiency increases linearly with increasing concentration of C-dots from 0.20 mg/mL to 0.80 mg/mL.

The self-illuminating CDs developed in this study would allow for researchers to take advantages of the benefits of CD, while eliminating the disadvantage of the need for external illumination. Future work should be done to isolate the conjugated complexes from the heterogenous samples to achieve concentrated self-illuminating CDs. Similar self-illuminating FNP could be developed by using a different FNP to replace CDs or using a different bioluminescent protein to replace Rluc. As BRET is dependent on the distance between BRET donor and BRET acceptor, the combination of multicolor C-dots and BRET technique could be used to measure protein-protein and protein-nucleic acid interactions. Consequently, the self-illumination of nanomaterials-based BRET system opens new door to the development of non-invasive biosensors.

## Methods

### Materials

Citric acid, urea, N-(3-Dimethylaminopropyl)-N’-ethylcarbodiimide hydrochloride (EDC), and N-Hydroxysuccinimide (NHS) were purchased from Sigma Aldrich (Oakville, Canada). Rluc and CTZ were purchased from Nanolight Technology (Pinetop, USA).

### Carbon dot synthesis

A microwave oven-assisted process was used to produce water-soluble CDs in this study^17^. Citric acid and urea in 1:1 ratio was added to excess distilled water resulting in a colourless, transparent solution. The solution was heated in a domestic 750 W microwave oven in 45 second intervals until the solution became dark brown clustered solid. The solid was cooled to room temperature, then frozen in the freezer overnight. The frozen sample was processed with freeze dryer (The Virtis Company, Gardiner, USA) for two days to isolate the C-dots.

### Bioconjugation of renilla luciferase onto carbon dot

Carbodiimide crosslinkers, EDC and EDC/NHS, were studied in the bioconjugation of *Renilla* luciferase and carbon dot, respectively. 10 μL of 20 mM EDC was added to CDs with different amounts, 0.10 mg/mL, 0.20 mg/mL, 0.40 mg/mL, 0.80 mg/mL, and 1.20 mg/mL, respectively. Three replicants of the described five samples were prepared. Distilled water was added to all samples to final volume of 400 μL, and the samples were incubated for 30 min at 37 °C. Each sample received 2 μM Rluc following further incubation at 37 °C. Each replicant was incubated for a different period of time: 1 hr, 6.5 hr, or 24 hr. After the final incubation, samples were used immediately for fluorescence study. The procedure was repeated for EDC/NHS-mediated conjugation; 10 μL of 20 mM NHS was added to the samples along with EDC and C-dots.

### Photoluminescence study

All fluorescence measurements were performed using PTI Fluorescence Master System (HORIBA Canada Inc.). Prior to fluorescence study of conjugated CD-Rluc complex, emission spectrum from 400 nm to 700 nm of CD and Rluc was measured. As CDs require external light source to become excited, the excitation wavelength was set to match the maximum emission of Rluc.

Emission spectrum for each sample was measured from 400 nm to 700 nm. External light source was turned off. Due to the short-lived nature of Rluc bioluminescence as shown in the supporting information (Fig. S3), step size was fixed at 10 nm, and integration was fixed at 0.1 s. In preparation for photoluminescence measurement, the measurements for the maximal bioluminescence were always taken in 1 min after 30 μM CTZ was added to samples.

## Supplementary information

Supporting information
