# Supplementary material for: Self-illumination of Carbon Dots by Bioluminescence Resonance Energy Transfer
Source: Sci Rep. 2019 Sep 24;9:13796. doi: 10.1038/s41598-019-50242-9 (PMC6760201; doi:10.1038/s41598-019-50242-9)
Supplement: Supplementary file 1 — Supporting information [file 41598_2019_50242_MOESM1_ESM.docx]

**Supporting Information**

**Self-illumination of Carbon Dots by Bioluminescence Resonance Energy Transfer**

Jisu Song^1^ and Jin Zhang^1,2,^*

^1^ School of Biomedical Engineering, University of Western Ontario, London, Ontario, N6A 5B9, Canada

^2^ Department of Chemical and Biochemical Engineering, University of Western Ontario, London, Ontario, N6A 5B9, Canada

* jzhang@eng.uwo.ca

**_

_Figure S1**

**Figure S1**. UV-vis absorption spectrum of CDs with different concentrations.

**Figure S2**


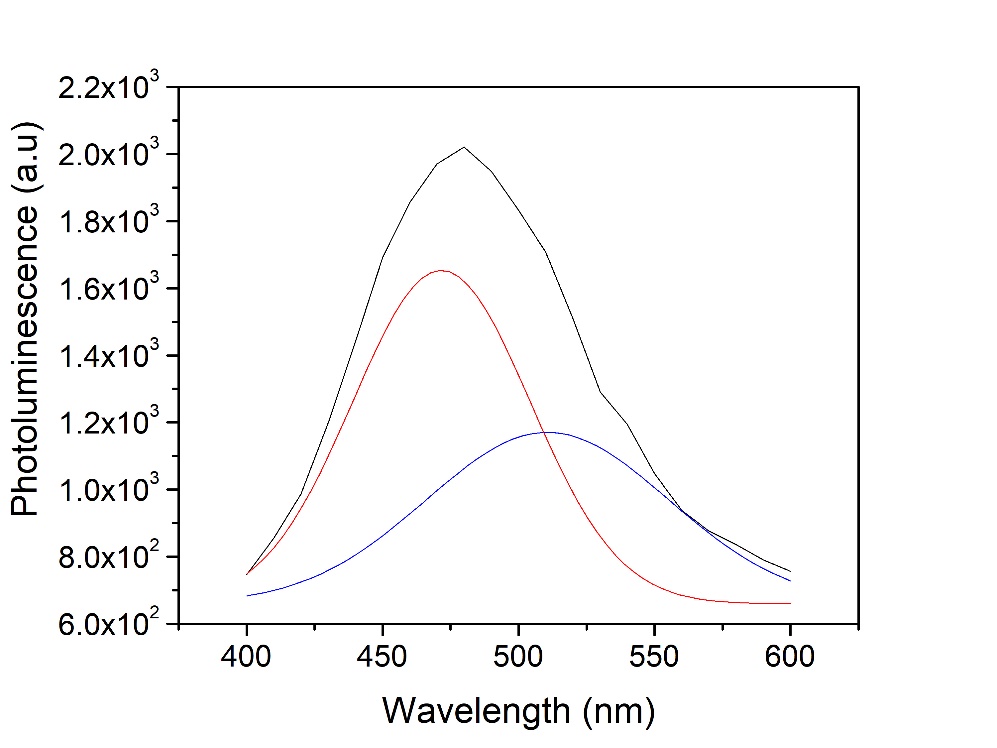

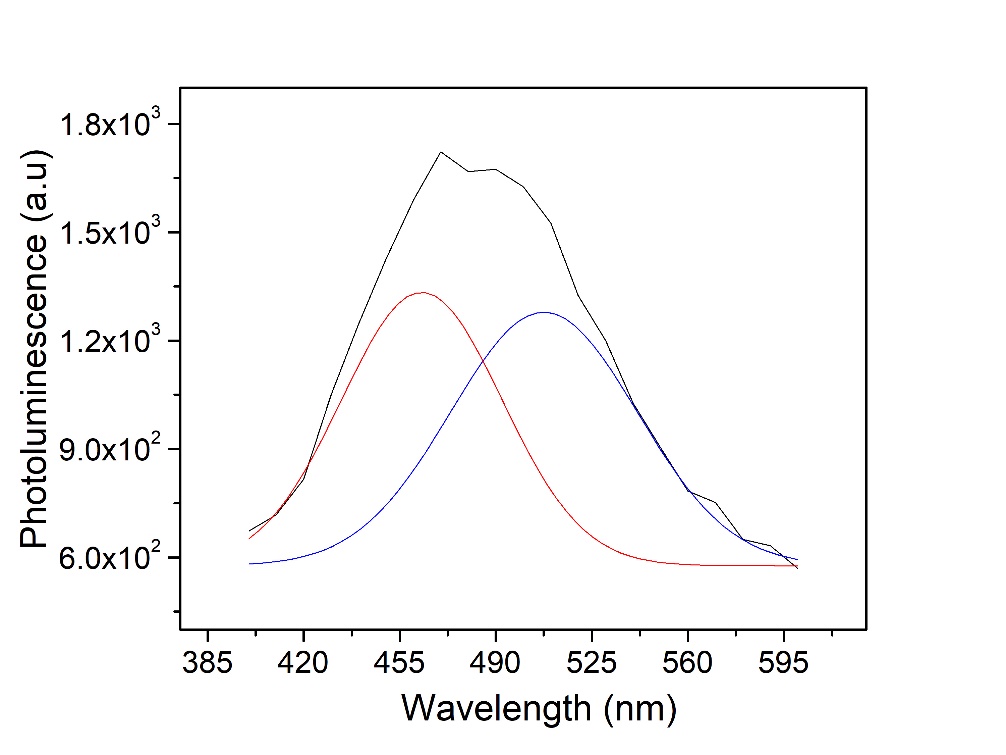


(a)

(b)

**Figure 2S.** (a) BRET spectrum of sample made through EDC-mediated conjugation of 0.40 mg/mL CDs and 2 μM Rluc for 6.5 hours. (b) BRET spectrum of sample made through EDC-mediated conjugation of 0.80 mg/mL CDs and 2 μM Rluc for 6.5 hours. Please note the black line represents the BRET profile of the sample; the red line and the blue line obtained by using multi-peak fitting are attributed to the bioluminescence of Rluc and the photoluminescence of CDs, respectively.

**Figure S3**

**
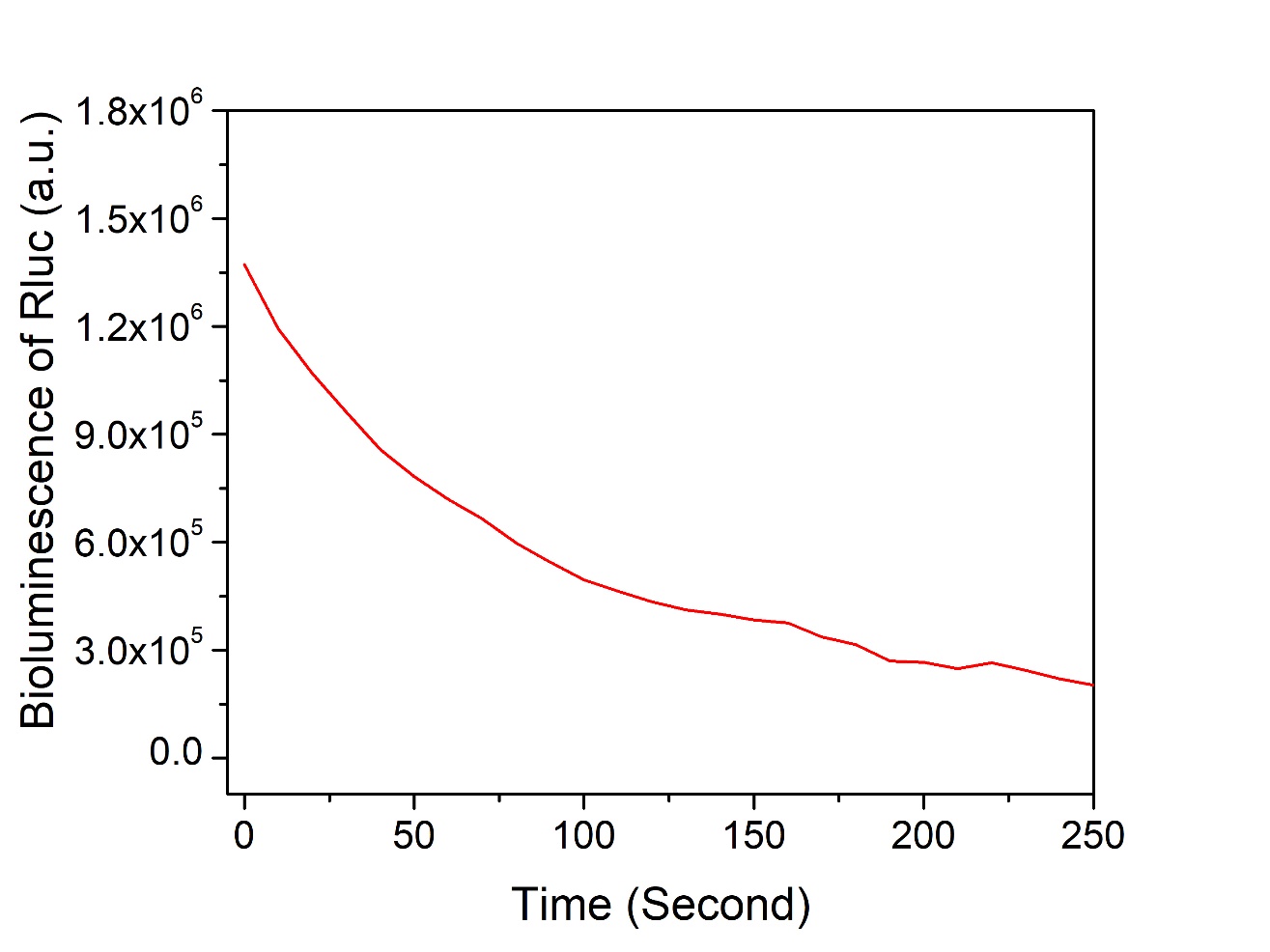
**

**Figure S3**. Photoluminescence of Rluc as a function of measurement time after introducing CTZ
